# Facile Preparation of Graphene Oxide-Enhanced Highly Crystalline Polyglycolic Acid Under Low-Temperature Crystallization Using Tin(II) 2-Ethylhexanoate and Its Application in Biomaterials

**DOI:** 10.3390/polym17233181

**Published:** 2025-11-29

**Authors:** Ho-Fu Chen, Jia-Wun Li, Kuo-Jen Ou, Shu-Yuan Yu, Jui-Hsin Wang, Chih-Chia Cheng, Yao-Hsuan Tseng, Yu-Hsun Nien, Chung-Feng Jeffrey Kuo, Chih-Wei Chiu

**Affiliations:** 1 Department of Materials Science and Engineering, National Taiwan University of Science and Technology, Taipei 10607, Taiwan; 2 Graduate Institute of Applied Science and Technology, National Taiwan University of Science and Technology, Taipei 10607, Taiwan; 3 Department of Chemical Engineering, National Taiwan University of Science and Technology, Taipei 10607, Taiwan; 4 Department of Chemical and Materials Engineering, National Yunlin University of Science and Technology, Yunlin 64002, Taiwan

**Keywords:** polyglycolic acid, ring-opening polymerization, Avrami equation, crystallinity, tin(II) 2-ethylhexanoate, graphene oxide

## Abstract

Polyglycolic acid (PGA), a biodegradable polymer with many potential applications, is primarily synthesized via the ring-opening polymerization of glycolide monomers. Here, the temperature sensitivity of the PGA crystallization kinetics is systematically analyzed using differential scanning calorimetry (DSC), Fourier-transform infrared (FTIR) spectroscopy, and X-ray diffraction (XRD). Regression analysis yields Avrami exponents (*n*) within the range 1.41–1.51, indicating that PGA forms lamellar crystals during crystallization from heterogeneous nucleation. The FTIR indicates that the PGA molecular chains alter their conformation during crystallization. XRD reveals that the crystallization rate and crystallinity of PGA closely correlate with the processing temperature. The heterogeneous nucleation of PGA can be optimized by incorporating suitable nucleating agents or regulating the surface roughness to improve the crystallization rate and quality. Polarized optical microscopy (POM) indicates that elevated temperatures increase the polymer chain mobility and free growth of crystallization nuclei, whereas lower temperatures promote the rapid formation of crystallization nuclei but impede growth. Graphene oxide (GO) has abundant surface functional groups, is an efficient heterogeneous nucleating agent, and promotes molecular chain alignment to increase both the crystallization rate and crystallinity. This GO-induced enhancement demonstrates a promising strategy for tailoring the thermal and mechanical properties of PGA for advanced biomedical applications.

## 1. Introduction

Polyglycolic acid (PGA), an important biodegradable polyester, has been widely used in biomedical applications in recent years. Its crystallinity strongly affects its degradation rate and mechanical performance, yet its highly temperature-sensitive crystallization behavior remains a major limitation that necessitates systematic kinetic analysis [1,2,3,4,5,6,7,8,9,10,11]. Recent advances in polymer nanocomposite engineering have demonstrated that the incorporation of inorganic nanofillers and two-dimensional materials can significantly enhance the crystallization kinetics of PGA [12] and improve the functional performance of biodegradable polymers [13,14,15]. In particular, graphene oxide (GO), owing to its large surface area and abundant oxygen-containing functional groups, has been shown to act as an efficient heterogeneous nucleating agent that promotes molecular chain alignment and accelerates crystal growth in polymer matrices [16,17]. However, its effect on the crystallization of PGA at low isothermal temperatures is still insufficiently understood [18,19]. In addition to its nucleation capability, GO is particularly suitable for use with PGA because both materials possess polar functional groups capable of strong hydrogen-bonding interactions. Specifically, the hydroxyl, epoxide, and carboxyl groups on GO provide abundant interfacial anchoring sites for the –CH_2_–COO– groups of PGA, which enables more efficient chain alignment compared to non-functional inorganic fillers. Moreover, the large nucleation surface area resulting from the two-dimensional morphology of GO is known to enhance crystallization in other polyesters such as PLA and PCL. These characteristics make GO a rational and effective choice for modulating the crystallization behavior of PGA, a highly crystalline aliphatic polyester with a high melting point and capable of strong intermolecular interactions [20,21,22,23]. Because of the strongly temperature-dependent crystallization behavior of PGA, slight variations in the crystallization temperature can significantly influence the crystallinity and crystal structure, thus making precise kinetic characterization essential [24]. Considering the strong influence of both the synthesis route and thermal history on the crystallinity of PGA, controlled studies of the crystallization kinetics are particularly important [25,26,27,28,29,30,31,32]. To present the fundamentals of PGA more systematically, the key structural and crystallization-related features are summarized here in a concise, integrated manner. The PGA possesses a highly regular backbone composed of repeating –CH_2_–COO– units, which enable tight chain packing and strong intermolecular interactions. These interactions—primarily hydrogen bonding and van der Waals forces—contribute to its high melting temperature, fast crystallization tendency, and sensitivity to thermal history. The crystallization of PGA is strongly affected by the temperature, degree of supercooling, molecular weight, residual moisture, and catalytic conditions during synthesis. Increased supercooling generally leads to reduced chain mobility, smaller crystals, and higher defect density, whereas mild supercooling favors more ordered lamellar structures. Collectively, these structural features make it necessary to precisely control the crystallization temperature to achieve the desired crystallinity and mechanical performance.

In polycondensation-based synthesis, residual water and temperature fluctuations can significantly affect the molecular weight and crystallinity. Elevated temperatures may also induce “retrograde bite,” in which the polymer chains revert to the cyclic glycolide, thereby limiting the achievable molecular weight and structural regularity. Furthermore, the presence of residual moisture in the glycolide melt can hinder chain packing and reduce the crystallinity, while the choice of catalyst and reaction conditions strongly influences the physicochemical properties of the resulting polymer [33]. Lee et al. [34] also demonstrated that the strong intermolecular interactions of PGA make its mechanical and thermal behavior highly sensitive to temperature changes, further underscoring the need to clarify its crystallization response under controlled thermal conditions. Similarly, supercooling reportedly strongly affects the size and ordering of PGA crystals [35]. These findings collectively indicate that the crystallization of PGA is highly sensitive to temperature, yet a systematic kinetic analysis at lower isothermal temperatures has not yet been reported. The available evidence suggests that the supercooling degree has a significant impact on the thermal properties of PGA, in addition to its effect on the crystal structure. A lower degree of order in the crystals causes the heat of fusion and melting temperature of PGA to decline, resulting in diminished thermal stability, which potentially limits its application at elevated temperatures. Therefore, controlling the crystallization conditions to minimize the degree of supercooling and defect formation is necessary to obtain high-performance PGA materials. In addition, suitable post-treatment techniques, such as annealing, can partially rectify crystal defects resulting from an elevated degree of supercooling, thereby enhancing the mechanical and thermal properties of the material. Annealing rearranges crystals, reduces the prevalence of defects, and enhances the crystallinity and overall performance of the material. The thermal behavior and crystallization kinetics data were successfully fitted to the Avrami equation [36,37,38,39], which describes the nucleation and growth mechanisms during the crystallization process. This not only illustrates the behavior of PGA under disparate crystallization conditions but also provides a theoretical foundation for further research on various crystallization mechanisms. The results are significant for the development of more energy-efficient chemical processes. For instance, the conditions of the ring-opening polymerization (ROP) reaction could be modified to optimize the crystallinity and thermal properties of PGA, thus reducing production costs and improving product quality. Furthermore, these data provide valuable insights that can inform the study of more complex materials, including copolymers of PGA, polymer blends, and polymer–inorganic composite materials.

Against this background, in addition to investigating the intrinsic crystallization kinetics of PGA under isothermal conditions, this study introduced GO as a heterogeneous nucleating agent to regulate the crystallization process. Owing to its large surface area and abundant oxygen-containing functional groups, GO was expected to facilitate chain alignment through hydrogen-bonding interactions, which would enhance the crystallization rate and crystallinity to ultimately improve the thermal and mechanical properties of PGA. This approach not only addresses the limitations associated with the crystallization behavior of neat PGA but also provides a promising pathway for developing advanced PGA-based biomaterials with tunable degradation profiles and enhanced functional performance. The systematic clarification of the crystallization behavior of PGA under low-temperature isothermal conditions and determination of the effective GO loading that significantly enhances heterogeneous nucleation provides new and useful insights.

## 2. Materials and Methods

### 2.1. Materials

Glycolide (≥99% assay) was used as the monomer in the preparation of polyglycolic acid (PGA), and 92.5% pure tin(II) 2-ethylhexanoate (Sn(Oct)_2_) was used as the catalyst (Sigma-Aldrich, Yongin, Republic of Korea). Graphene oxide (GO, 40% oxygen content) was purchased from Enerage Inc., Yilan, Taiwan. Hexafluoroisopropanol was purchased from Echo Chemical Co., Toufen, Taiwan.

### 2.2. Ring-Opening Polymerization of Polyglycolic Acid

**Figure 1:**
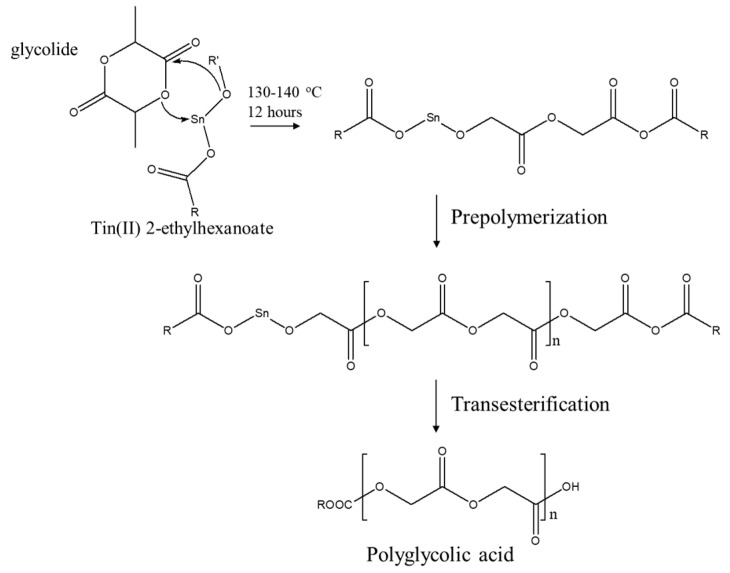
Synthesis of PGA through the ROP reaction of glycolide monomers.

Figure 1 illustrates the synthesis of PGA through the ROP reaction of the glycolide monomer [40]. The ROP of glycolide to produce PGA is catalyzed by Sn(Oct)_2_, which initially forms a coordination compound with the glycolide monomer to reduce the ring strain within the glycolide molecule and facilitate the subsequent ring opening. The figure depicts the coordination structure of the glycolide monomer with Sn(Oct)_2_. The cyclic structure of glycolide becomes unstable owing to its coordination with Sn, thereby facilitating ring opening, whereupon a new active center is formed. This active center is a coordinated structure of Sn with an open glycolide monomer that can further attack other glycolide monomers. The figure depicts the chain growth process, wherein the structure shown represents the aforementioned phenomenon. Subsequently, the opened glycolide molecule reacts with another glycolide monomer, whereby the ring continues to open and integrate to form the polymer chain. The repetition of this process results in the gradual formation of long-chain PGA. Eventually, following a series of chain-growth steps, PGA, which consists of repeated glycolide monomer units, is formed. The process may continue under suitable reaction conditions until the reaction is terminated or all monomers have been consumed. In Figure 1, the Sn atom, situated at the center, forms coordination bonds with the oxygen atoms of glycolide. The glycolide ring is already partially opened and coordinated with Sn. The R group represents a portion of the PGA chain that is in the process of forming. The dashed coordination bonds represent the coordination interaction between Sn and the oxygen atoms of glycolide. The mechanism of PGA formation entails the coordination of Sn(Oct)_2_ with glycolide monomers to form an active center. The glycolide then opens to form a new active center that attacks additional glycolide monomers, thus continuing the ROP. These steps are repeated until long-chain PGA is formed. The coordination interaction between Sn and glycolide drives each step of the process and enables efficient ring opening and polymerization of the glycolide monomers to form PGA.

Glycolide polymerization was performed in a 100 mL three-necked flask equipped with a nitrogen (N_2_) inlet/purge device, a vacuum controller, and a mechanical stirrer. The temperature and pressure were fixed according to preliminary optimization to ensure stable polycondensation. Under a nitrogen atmosphere, the flask was charged with glycolide monomer, and a silicone oil bath was used for heating. The temperature was increased to 130–140 °C, at which the glycolide monomer melts into a homogeneous liquid. Subsequently, the system pressure was reduced to 100 Torr to eliminate low-boiling impurities that could potentially interfere with the polymerization process. Following a 30 min interval, the system was replenished with dry nitrogen (N_2_). Afterward, 0.5 wt% of the Sn(Oct)_2_ catalyst, calculated based on the mass of glycolide monomer, was introduced into the reactor, and the reaction was allowed to proceed for approximately 12 h. The reaction was terminated by gradually lowering the temperature to ambient levels, following which the system was evacuated under vacuum for 12 h at 40 °C.

### 2.3. Fabrication of GO/PGA Nanocomposites

An appropriate amount of GO was added to hexafluoroisopropanol (HFIP) to ensure complete dispersion within the solvent system. The reported GO concentration (wt%) refers to the solid GO content after solvent removal, and the loading amount was verified by weighing the dried GO prior to dispersion. To prevent agglomeration of GO nanosheets and promote uniform exfoliation, a preliminary dispersion step was conducted via ultrasonication for 15 min. This step facilitated the breakup of GO aggregates through acoustic cavitation. The GO was then subjected to a secondary dispersion step using an ultrasonic processor under controlled conditions (amplitude: 10%, pulse-on time: 10 s, pulse-off time: 3 s, and total processing time: 30 min), which ensured the formation of a stable and homogeneously dispersed suspension. The resulting GO dispersion was subsequently introduced into the PGA solution under continuous stirring to facilitate strong interfacial interactions and hydrogen bonding between the functional groups of GO and the PGA chains. The mixture was then cast and dried in a vacuum oven to remove residual solvent to ultimately yield a well-integrated GO/PGA nanocomposite with uniformly distributed GO nanosheets embedded within the polymer matrix.

### 2.4. Structural Characterization by FTIR

The FTIR spectra of the PGA samples were recorded on an FTIR spectrometer (Perkin Elmer FTS-1000, Hopkinton, MA, USA). To reduce the impact of moisture on the samples, they were dried for 12 h. The scanning range spanned 4000 to 200 cm^−1^. The FTIR spectra were collected in transmission mode using solution-cast films prepared from HFIP to obtain a uniform sample thickness. Each spectrum was acquired with 32 scans at a resolution of 4 cm^−1^. Peak assignment and interpretation followed standard polymer FTIR references [41,42], which include the characteristic vibrational modes of PGA such as C=O stretching (~1745 cm^−1^), C–O–C asymmetric stretching (~1250–1180 cm^−1^), and CH deformation (~1450 cm^−1^).

### 2.5. XRD Analysis

The XRD patterns were obtained using a Thermal ARL X’tra X-ray diffractometer. The X-rays were generated using a sealed tube with a wavelength of 0.154 nm (CuKα). Bragg’s law (*nλ* = 2*dsinθ*) was employed to calculate the interplanar spacing [43], where *θ* represents the angle between the incident ray and the scattering plane, *n* represents an integer denoting the order (*n* = 1, 2, or 3), and *λ* represents the wavelength of the X-ray. The patterns were recorded over a 2*θ* range of 10–40°, with a step size of 0.2° and a fixed counting time of 10 s.

### 2.6. DSC Thermal Analysis

The DSC (Perkin–Elmer DSC 6) was employed to thermally characterize PGA, including its melting and crystallization behaviors. The DSC experiments were conducted with a N_2_ flow rate of ≈20 mL min^−1^ following standard DSC procedures for polymer crystallization [44]. The basic DSC procedures followed the general guidelines of ASTM E793 and ASTM E794, while the melt–erase–isothermal protocol and specific heating/cooling rates were adapted from standard crystallization kinetic studies to ensure complete melting and stable isothermal crystallization behavior. A sample weighing ≈5 mg was sealed in an aluminum foil pouch under a nitrogen atmosphere. The non-isothermal DSC program consisted of six steps, structured to erase the thermal history and determine the crystallization onset temperature.

(A) Equilibration: sample stabilized at 25 °C for 1 min.
(B) Heating: 25 → 235 °C at 10 °C min^−1^.
(C) Isothermal erase: 235 °C for 5 min to remove the thermal history.
(D) Cooling: 235 → 25 °C at 30 °C min^−1^.
(E) Re-equilibration at 25 °C for 1 min.
(F) Heating: 25 → 235 °C at 5 °C min^−1^.

To completely melt the PGA samples and to eliminate any residual crystals, the samples were heated at a rate of 3 °C min^−1^ from 25 to 235 °C, which is slightly above the observed melting temperature, *T_m_*. This completely removed the crystalline memory prior to isothermal crystallization in the thermal history of the sample. The appropriate isothermal crystallization temperatures (T1–T5) of the samples were determined using DSC thermograms (cooling scans) under non-isothermal crystallization conditions. Five temperatures (175, 176, 177, 177.5, 185 °C) were selected within the crystallization window that had been determined experimentally.

These temperatures lie within the experimentally identified optimal crystallization range (175–178 °C). Furthermore, the tests revealed that the crystallization rate was markedly slow when the temperature exceeded 180 °C. Therefore, we surmised that no crystals would form in the samples during the rapid cooling process until the temperature dropped to the specified crystallization temperature. The isothermal crystallization process comprised the following six stages:

(A)–(C) followed the same melting and erase steps as above.
(D) Rapid cooling: 235 °C → T1–T5 at 30 °C min^−1^.
(E) Isothermal hold: 90 min at T1–T5.
(F) Heating: T1–T5 → 235 °C at 5 °C min^−1^.

The DSC instrument used in this study has an enthalpy precision of approximately ±1% and a temperature accuracy of ±0.1 °C, which ensured that the differences among the crystallization temperatures within the range 175–178 °C exceed instrumental uncertainty. Crystallinity was calculated using the standard DSC enthalpy method, where Xc = (ΔHm/ΔH^0^) × 100%, with ΔHm representing the measured melting enthalpy and ΔH^0^ = 206 J g^−1^ taken from the literature [44] as the enthalpy of 100% crystalline PGA. The original DSC data for each crystallization temperature were obtained from three independent measurements, and the reported values represent the average of these triplicate runs.

All syntheses and DSC measurements were performed in triplicate, and the reported values represent the mean ± standard deviation. All relevant thermograms are shown in Section 3 for clarity.

## 3. Results and Discussion

### 3.1. Structural Characterization of Polyglycolic Acid Synthesized by Ring-Opening Polymerization

**Figure 2:**
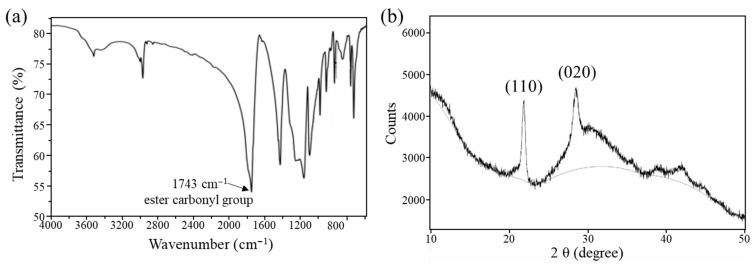
(**a**) FTIR spectra of PGA in the wavelength range of 4000–650 cm^−1^. (**b**) XRD pattern of PGA.

This synthesis was performed on multiple occasions with reproducible results, thereby confirming the reliability of the procedure. To confirm that the polymer synthesized using the aforementioned method was PGA, the prepared samples were analyzed using FTIR spectroscopy and XRD. The structure was verified by monitoring the absorption bands that correspond to the different functional groups of PGA. The FTIR spectrum of PGA, shown in Figure 2a, was recorded in the range of 4000 to 650 cm^−1^. The absorption band at 3541 cm^−1^ is attributable to hydrogen-bonded O–H stretching vibrations, which likely originate from trace moisture or hydroxyl (OH) groups, while that at 2963 cm^−1^ is attributable to the C–H stretching vibrations of alkyl groups (CH_3_ or CH_2_), such as in alkanes or compounds with extended carbon chains. The 1742 cm^−1^ peak is characteristic of the C=O stretching vibration of the ester group in PGA. The 1419 cm^−1^ peak corresponds to C–H bending vibrations of methyl (CH_3_) or methylene (CH_2_) groups, which are typically present in alkanes or aliphatic compounds. The 1153 cm^−1^ peak corresponds to the C–O stretching vibration, which may be attributed to C–O bonds in alcohols, ethers, or, in this case, esters. The C–O–C stretching vibration, commonly found in ethers or esters, was observed at 1089 cm^−1^. The peaks at 971, 901, 809, 720, and 592 cm^−1^ correspond to C–H bending vibrations in aromatic rings. A comprehensive analysis of the spectrum allowed us to identify the following structural units of PGA: the ester group was confirmed by the presence of a carbonyl (C=O) peak at 1742 cm^−1^; the alkyl group was identified by the C–H stretching vibration at 2963 cm^−1^ and the C–H bending vibration at 1419 cm^−1^; and the alcohol or ether group was identified by the C–O and C–O–C stretching vibrations at 1153 and 1089 cm^−1^, respectively. This combination of spectral data confirmed that the ROP of glycolic acid yielded PGA as an organic polymer containing ester, alkyl, and alcohol or ether groups. In addition, the polymer was analyzed using XRD to examine its crystal packing, as shown in Figure 2b. The XRD patterns of the PGA samples contain diffraction peaks within the 2θ = 10–50° range, corresponding to the various crystal planes of PGA, particularly the 110 and 020 planes. This indicated the presence of crystalline regions in the samples. The diffraction peak of the 110 plane at ≈20° is distinctive and indicates the existence of substantial crystalline regions within the samples, suggesting that the PGA molecular chains are highly ordered in this direction. The intensity and width of this diffraction peak can be used to ascertain the dimensions and number of crystalline regions. The presence of strong and narrow peaks indicates larger crystalline regions and higher crystallinity, which benefit the mechanical strength and durability of PGA. Specifically, higher crystallinity can enhance the strength and rigidity of the material, making it less susceptible to deformation under stress. Moreover, the presence of larger crystalline regions can enhance the resistance of the material to wear and chemical degradation. The diffraction peak of the 020 plane between 25° and 30° has a distinct reflection that corroborates the crystallinity of PGA and an ordered, alternating configuration of molecular chains. The presence of the 020 plane indicates the existence of highly ordered crystalline regions in PGA. Furthermore, the intensity and width of the reflection peak of the 020 plane offer insight into the characteristics of the crystalline regions. The intensity of the 020 diffraction peak is indicative of the extent of the crystalline regions present in the PGA samples, which has a considerable impact on the physical properties of the material. A more intense 020 peak indicates a more uniform arrangement of molecular chains in different directions, which increases the isotropy of the material and results in similar physical properties in all directions. The background signals observed on the XRD pattern suggest the existence of amorphous regions with a lack of ordered molecular configuration within the PGA samples, in contrast to the crystalline regions. The presence of amorphous regions may impact the flexibility and degradation behavior of PGA, yet they also offer certain advantages in terms of machinability. Amorphous regions facilitate the manipulation and shaping of the material during processing while also imparting a degree of toughness, enabling it to absorb greater energy under stress without fracturing. The crystallinity of the PGA samples was determined by analyzing the intensities of the XRD peaks and background signals. A high degree of crystallinity is typically associated with the enhanced mechanical properties of the material; however, this may also reduce the degradation rates. The PGA with a high degree of crystallinity exhibits enhanced strength and durability, which renders it well-suited for applications that demand long-term stability, such as medical implants and sutures. Conversely, low crystallinity typically leads to accelerated degradation rates, although it may also have a detrimental impact on the mechanical properties. The PGA with low crystallinity is more suitable for applications that require rapid degradation, such as drug delivery systems.

### 3.2. DSC Analysis—Non-Isothermal Crystallization

**Figure 3:**
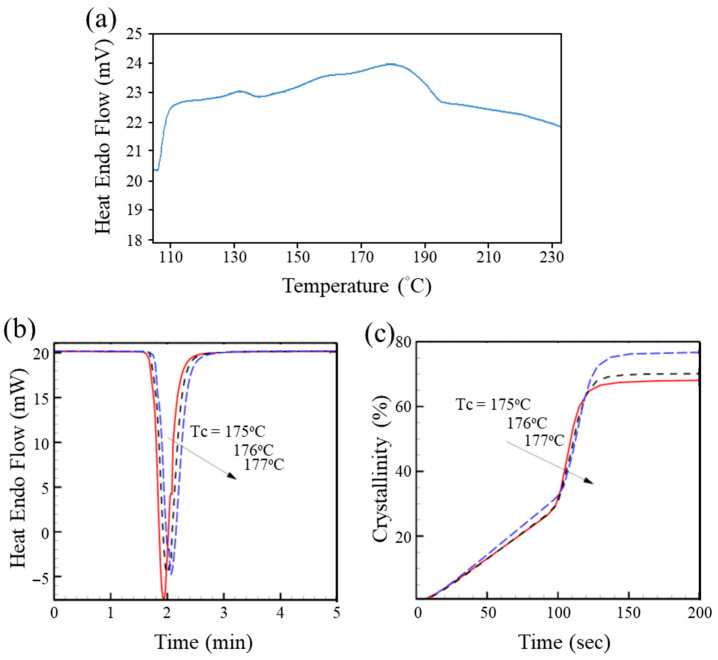
(**a**) DSC thermogram (heating scan) of synthesized PGA (scan range: 25–235 °C; scan rate: 10 °C min^−1^). (**b**) Heat endo flow measurements during isothermal crystallization of PGA at varying *T_c_*. (**c**) DSC crystallinity during the isothermal crystallization process of PGA over time.

The DSC was used to determine the crystallization temperature (*T_c_*), crystallization time, and melting temperature (*T_m_*) of the samples. The samples were prepared under non-isothermal crystallization conditions, and the values of the aforementioned properties were obtained from the exothermic peaks. Figure 3a depicts the DSC thermogram of PGA acquired at a heating rate of 10 °C min^−1^. The thermal scan spanned a temperature range of 25–235 °C, and the endothermic peak between 175 and 177 °C indicates that significant thermal effects were induced by alterations in the crystal arrangement. Accordingly, this narrow temperature range was selected for subsequent isothermal crystallization studies. Figure 3b presents the heat flow data during the crystallization process at different temperatures in this range (*T_c_* = 175~177 °C), whereas Figure 3c shows the relative crystallinity, *X*(*t*), as a function of time. The definition of *X*(*t*) is as follows:

(1) $$X(t)=\frac{\Delta H(t)}{\Delta H(\infty )}$$

As shown in Figure 3b,c, the crystallization kinetics slightly accelerate at relatively low temperatures. This phenomenon is consistent with the results of previous studies conducted in different process temperature ranges [20]. However, our findings differ from previous findings in two ways. The final crystallinity is highly sensitive to isothermal crystallization temperatures between 175 °C and 177 °C, with the crystallinity dropping from 77% to 68%, a difference of ≈10% within this narrow temperature range. Table 1 indicates that the relative crystallinity of ROP PGA reaches its maximum value within the temperature range of 176.5–177 °C. When the crystallization temperature was increased by an additional 0.5 °C, the decline in crystallinity was notable. These results indicate that an isothermal crystallization temperature between 176.5 and 177 °C may be optimal if the main goal is for the material to be as strong as possible.

**Figure 4:**
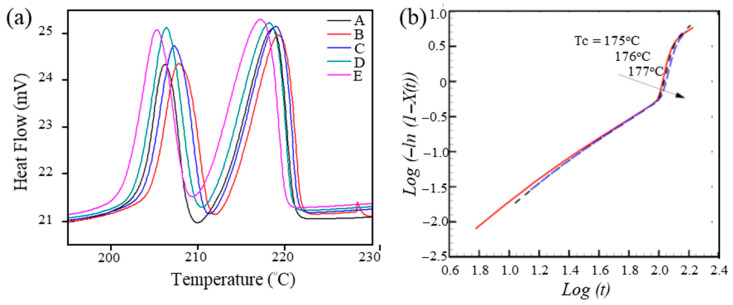
(**a**) Plot of *Log*(*−ln*(1 − *X*(*t*))) as a function of *Log*(*t*) at varying *T_c_*. (**b**) DSC curves (second heating scan) of PGA for various crystallization temperatures to 235 °C at a heating rate of 5 °C min^−1^.

In this study, we focused on the crystallization temperature range of 175–177.5 °C to determine the melting points of the crystals and compare the melting enthalpies. The heating scans in Figure 4a enabled the degradation or crystallization of PGA over time to be examined as a function of temperature. The dual melting peaks may correspond to α and β crystalline forms of PGA, as reported in earlier structural studies that identified these two polymorphic modifications [45]. The observed trend in curve variations indicates that the degradation or crystallization rate of PGA increases with temperature, which is important for optimizing temperature control and process regulation of PGA in practical applications. The final crystallinity in percent (%) reaches its maximum at 176.5 °C and 177 °C. The Avrami exponent (*n*), which varies between 1.41 and 1.51, provides insight into the mechanism and nucleation characteristics of the crystallization process. The obtained Avrami exponent (*n* ≈ 1.5) is consistent with heterogeneous nucleation and two-dimensional lamellar growth, which aligns with previously reported values for PGA crystallization [46]. The crystallization rate constant (*K_c_*) exhibits a maximum at 177.5 °C, indicating a notable alteration in the crystallization rate. The half-crystallization time (t1/2), which ranges from 111 to 116 s, varies minimally in the time required to achieve 50% crystallinity at varying crystallization temperatures.

In conclusion, the impact of the crystallization temperature on PGA reveals that the highest final crystallinity is attained at 176.5 and 177 °C, indicating optimal crystallization efficiency. This indicates that the crystallization process of PGA is more efficient at these temperatures. The variation in the crystallization rate indicates that, despite the lower final crystallinity observed at 177.5 °C, the crystallization rate constant is the highest, suggesting an accelerated crystallization rate at this temperature. However, the crystallinity may be limited by other factors. The Avrami exponent (*n*) indicates that the nucleation and growth processes of PGA at these temperatures are predominantly heterogeneous nucleation and lamellar growth. The crystallization rate constant (*K_c_*) and half-crystallization time (t1/2) show notable discrepancies in the crystallization rates and the time required to attain 50% crystallinity at varying temperatures, respectively, underscoring the considerable influence of temperature on the crystallization process.

After the PGA materials were crystallized at varying temperatures, the melting points of the crystals were determined by heating them to 235 °C. This enabled a comparison of the enthalpies of fusion, which are summarized in Table 2. Figure 4b shows the presence of two endothermic peaks at 207 and 218 °C upon heating to 235 °C, indicative of two distinct crystal structures in PGA, which ultimately contribute to the heat absorption during the melting process. This study revealed that while the crystallinity at *T_c_* = 177 °C is higher than at *T_c_* = 177.5 °C, the relatively high melting enthalpy at *T_c_* = 177.5 °C has a significantly more pronounced effect than samples crystallized at lower temperatures. This phenomenon can be attributed to the competing effects of chain mobility and nucleation density: although higher temperatures (e.g., 177.5 °C) enhance the chain mobility and accelerate crystal growth, they simultaneously reduce the density of effective nuclei, leading to lower overall crystallinity compared with that at 177 °C. Evidently, the crystals melting at 218 °C formed more compact structures, which necessitated greater energy input to release the polymer. The results indicate that a higher *T_c_* results in the formation of crystals with greater density. The crystallinity and distribution of the material are of great significance regarding the physical strength of PGA. The aforementioned study illustrates that the dynamic crystallization behavior of PGA polymers is significantly influenced by both the polymer synthesis method and the selected crystallization temperature. As is clear from Table 2, the ultimate crystallinity values are contingent upon the synthesis conditions of the polymer, including the selection of the reaction catalyst, the reaction method (whether low-molecular-weight molecules are removed), and the temperature during crystallization. It is exceedingly difficult to completely eliminate low-molecular-weight molecules in processes such as melt/solid polycondensation or solvent/dispersion polymerization. These small molecules act as plasticizers in PGA polymers by interfering with the formation of solid packing by disrupting interactions between polymer chains. This adversely affects the mechanical properties and crystallization behavior of the material. The crystallization process can be described as an essentially thermodynamic phase transition, yet it is also subject to influence from kinetic mass-transfer processes, which depend on the interactions between and within the polymer molecules. For example, the movement of polymer chain segments, the formation of crystallization nuclei, and the growth rate all influence the final crystallinity and crystallization rate. Accordingly, the crystallization rate is contingent upon both the molecular orientation and melt viscosity of the polymer, both of which are inextricably linked to the molecular weight and polymer dispersity index (PDI) of the polymer. Although the relationship between molecular weight distribution and crystallization behavior is well established in the literature, we note that the PDI of the synthesized PGA (e.g., via GPC) was not directly measured in our study. Therefore, our discussion of the PDI serves only as a general theoretical explanation rather than an experimentally validated parameter in this work. A detailed quantitative analysis of the PDI and its effects on the crystallinity and mechanical properties of PGA is planned for our future study.

These findings show that the final crystallinity of PGA synthesized through ROP is superior to that of PGA obtained via alternative synthesis techniques. This may be because ROP enables the molecular weight and PDI of the polymer to be more precisely controlled, which allows more efficient crystallization. Furthermore, the selected crystallization temperature has a substantial impact on the formation of crystals and the kinetics of PGA. At elevated crystallization temperatures, the higher kinetic energy of the polymer chains enables enhanced flexibility of motion and sufficient time for optimal crystallographic arrangements to be achieved. The high-energy environment allows the polymer chains to rapidly reorganize to form more ordered crystalline structures and thereby enhance the final crystallinity. Conversely, at lower temperatures, despite the higher rate at which crystallization nuclei are formed, the comparatively limited mobility of the polymer chains lowers the crystallization rate and potentially decreases the final degree of crystallinity. Therefore, exercising precise control over the crystallization temperature and polymerization conditions is imperative to obtain high-performance PGA materials. In conclusion, the crystallinity and crystallization rate of PGA are significantly influenced by a range of conditions that arise during polymerization. The selection of appropriate synthesis techniques and crystallization conditions can markedly improve the material properties of PGA. The subsequent discussion presents detailed data and analyses to support these observations and conclusions. In addition, the effect of incorporating the inorganic filler (GO) into the PGA matrix on the crystallization behavior and thermal properties was evaluated under identical conditions, as summarized in Table 2. The results indicate that when the GO content reached 2 wt%, the melting temperature (*T*_m_) increased, indicating an enhancement in crystallinity. This improvement can be attributed to the heterogeneous nucleation effect of GO, where its large specific surface area and abundant surface functional groups facilitate the oriented alignment of PGA molecular chains, thereby accelerating nucleation and promoting crystalline growth.

However, increasing the GO content beyond 2 wt% had the effect of lowering *T*_m_. This phenomenon is likely due to the aggregation of excessive GO, which disrupts the orderly packing of PGA chains and hinders the growth of crystalline lamellae, impeding the efficiency of the crystallization process. The calculated crystallinity of the GO/PGA nanocomposites confirms this trend: the crystallinity values at 1 wt%, 2 wt%, and 3 wt% GO loading were 78%, 79%, and 76%, respectively, consistent with the results of the melting temperature analysis. These findings demonstrate that 2 wt% GO provides the optimal nucleation density, which leads to the highest crystallinity in the PGA matrix. This trend is also reflected in the ΔH*_f_* values listed in Table 2, where the 2 wt% GO sample exhibits the highest fusion enthalpy in correspondence with its maximum crystallinity.

### 3.3. Morphology of Polyglycolic Acid in Different Crystallization States Using POM Crystal Image Analysis

**Figure 5:**
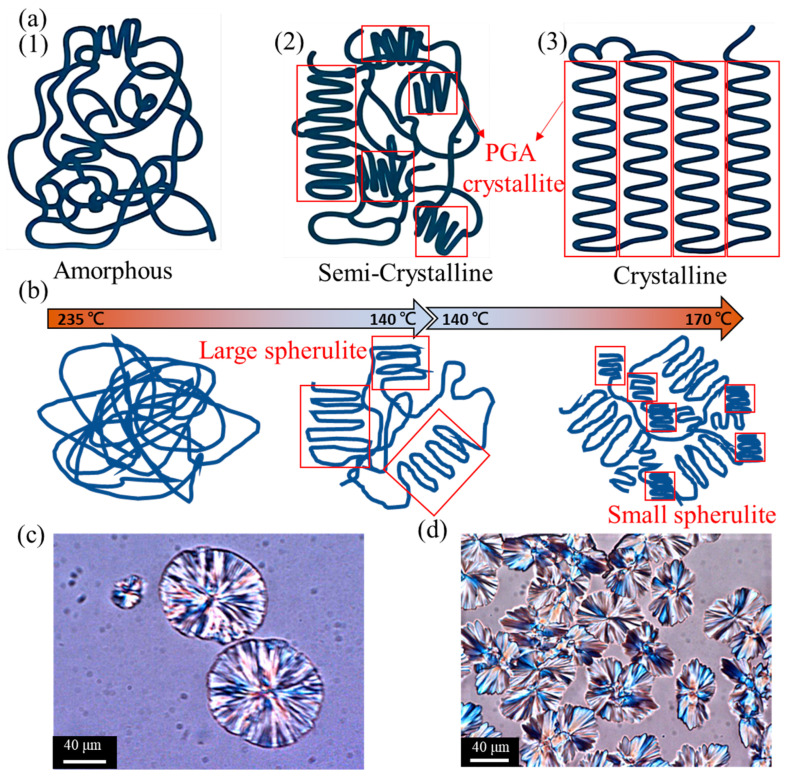
(**a**) Morphology of polymers in different crystalline states: (1) amorphous, (2) semi-crystalline, and (3) crystalline. (**b**) Schematic of the crystallization process, where PGA is rapidly cooled from a high-temperature molten state to the initial crystallization temperature, resulting in slow nucleation, followed by gradual temperature decrease to 160–170 °C to promote rapid crystal growth. (**c**) POM image analysis of the early stages of PGA crystallization (scale bars = 40 μm). (**d**) POM image analysis of the more advanced crystallization state of PGA (scale bars = 40 μm).

**Figure 6:**
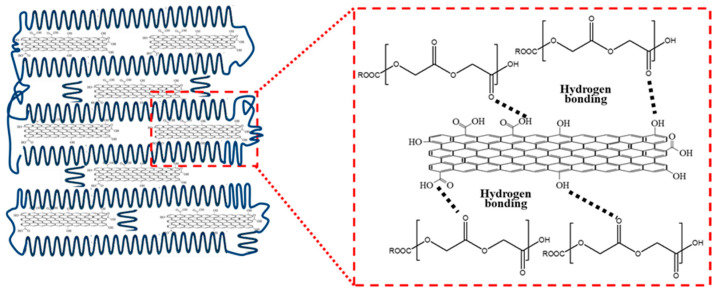
Schematic illustration of graphene oxide (GO) surface functional groups forming hydrogen bonds with PGA chains and acting as heterogeneous nucleation sites for PGA crystallization.

Figure 5a illustrates the morphological characteristics of the PGA polymer in three distinct crystallization states: amorphous, semi-crystalline, and crystalline. In the amorphous state, the disordered molecular chains impart flexibility to PGA as the chains can move freely. However, this disorder adversely impacts the mechanical strength and thermal stability. Furthermore, amorphous regions are susceptible to water absorption, which accelerates the hydrolytic degradation of PGA. Semi-crystalline PGA is composed of both amorphous and crystalline regions, where the latter provide structural strength and stability and the former offer a certain degree of flexibility. In this state, PGA is moderately degradable, which renders it suitable for medical applications such as surgical sutures and biodegradable implants. In the crystalline state, the highly ordered molecular chains of PGA give rise to a stable crystal structure; crystalline PGA has the highest mechanical strength and thermal stability, but with a flexibility tradeoff. Highly crystalline PGA is typically suitable for applications that require high strength and durability, such as bone fixation devices. The tightly packed crystalline regions also reduce the degradation rate. Figure 5b presents a schematic of the distinct stages in the crystallization process of PGA. Figure 5c,d show POM images of the initial and more advanced stages of crystallization, respectively. Figure 5c depicts a multitude of discrete spherulitic structures, which display radial growth patterns emanating from their centers. These spherulites indicate the commencement of crystallization following the formation of nuclei and initial growth. The restricted number and scattered distribution of the spherulites suggest that the crystallization nuclei formed slowly, which allowed each nucleus to expand freely to a considerable size. This stage of crystallization likely occurs at higher temperatures, as elevated temperatures facilitate polymer chain mobility and promote the growth of crystallization nuclei. Figure 5d depicts densely distributed spherulitic structures in close proximity to one another to form a highly crystalline region. This represents the subsequent stage of crystallization, wherein the number of crystallization nuclei has increased, and each nucleus has undergone a certain degree of growth before interacting with other nuclei. The higher rate at which crystallization nuclei form and the lower growth rate result in the formation of numerous small, densely arranged spherulites. This situation is likely to occur at lower temperatures. The initial crystallization phase in Figure 5c is distinguished by the presence of a limited number of large spherulites, which is typical for crystallization at elevated temperatures. By contrast, the subsequent crystallization stage in Figure 5d is characterized by the presence of numerous small, densely packed spherulites, which is characteristic of low-temperature crystallization. As time progresses, the number of crystallization nuclei increases, and spherulites grow and begin to contact one another, increasing the degree of crystallinity. The crystallization rate depends on the mobility of the polymer chains and the formation and growth rates of the crystallization nuclei. Elevated temperatures facilitate rapid polymer chain mobility and free growth of the crystallization nuclei, whereas lower temperatures promote the rapid formation of crystallization nuclei but impede subsequent growth. Understanding these crystallization behaviors is critical for the application of PGA as a material, as it allows for the optimization of PGA processing and performance to meet the requirements for specific applications. However, as illustrated in Figure 6, the incorporation of GO fundamentally alters the crystallization behavior of PGA. Owing to its abundant surface functional groups (e.g., hydroxyl and carboxyl groups), GO can form strong hydrogen bonds with the carbonyl and ether oxygen groups along the PGA chains. These molecular interactions facilitate the adsorption and alignment of PGA chains at the GO interface to effectively lower the nucleation energy barrier. Consequently, GO acts as a highly efficient heterogeneous nucleating agent that promotes the formation of ordered crystalline domains, thereby accelerating the crystallization rate and significantly increasing the overall crystallinity of PGA. This enhanced nucleation effect leads to the development of a more densely packed and thermodynamically stable crystalline structure.

### 3.4. Crystallization Behavior Regression Based on the Avrami Equation

The variation in the DSC endotherm data over time was fitted to the well-established Avrami equation to assess the crystallization process:

(2) $$1-X(t)=1-\frac{\Delta H(t)}{\Delta H(\infty )}=1-\frac{A_{t}}{A_{\infty }}=\exp(-K_{c}t^{n})$$

where *X*(*t*) represents the relative crystallinity as a function of time, *t*; *H*(*t*) denotes the measured heat flow as a function of time; *H*(∞) signifies the measured heat flow when crystallization is complete; *A_t_*/*A*_∞_ is the ratio of endothermic heat flow areas obtained from DSC, which is equivalent to *H*(*t*)/*H*(∞); *K*_c_ is the crystallization constant; and *n* is the Avrami exponent. Notably, *H*_0_ for 100% crystallization has been reported to be 206 J g^−1^ [47], which was evaluated in comparison with *H*(∞) at different isothermal crystallization temperatures. Equation (2) can be rearranged to yield Equation (3):

(3) $$Log\left[-\ln\left(1-X(t)\right)\right]=n\log t+\log K_{c}$$

Equation (3) represents a linear regression model applied to the DSC curves. Subsequently, the Avrami exponent, *n*, and the crystallization rate constant, *K*_c_, can be evaluated. The results are presented in Table 3. In this study, *n* ranged from 1.89 to 2.11, and *K*_c_ ranged from 2 × 10^−4^ to 7 × 10^−5^ as the crystallization temperature decreased from 177 to 175 °C. The crystallization process indicated that PGA developed lamellar crystals with uniform nucleation and thermodynamic phase transition, as evidenced by *n* ~ 2. Figure 4b shows regression plots (log[−ln(1 − *X*(*t*))] as a function of log(*t*) for *T_c_* ranging from 177 to 175 °C. The three curves displayed a linear relationship during the initial stages of crystallization but subsequently exhibited nonlinear behavior after reaching half of the relative crystallinity. As indicated in Table 1, from the perspective of the kinetic model, *n* was the highest at all crystallization temperatures, yet its corresponding *K*_c_ was not the lowest [48,49,50,51]. This indicates that the crystallization kinetics were constrained by mass transfer, as the correlation between *n* and *T_c_* did not follow a straightforward linear increase or decrease but instead fluctuated with temperature, increasing at some points and decreasing at others. Table 3 summarizes the results of related work to provide a reference point for the above PGA synthesis methods and their corresponding thermal properties. In this study, the synthesis methods in the table were assessed, with Method 7 (ROP) ultimately selected as the experimental model. This selection was based on the following characteristics. Thermal properties: Method 7 yielded PGA with a higher melting point (*T*_m_) of 218 °C and higher crystallinity (68–77%) than PGA prepared using other methods. This makes it particularly suitable for high-temperature resistance applications owing to its enhanced thermal stability and mechanical properties. Reaction conditions and time: Method 7 operates at lower reaction temperatures (130–140 °C) and for a shorter duration (12 h) than Methods 1 and 2, which operate at a high temperature (190 °C) and for a long duration (20 h), as well as Method 6, which operates at high temperatures (170–200 °C) for a long duration (20 h). Method 7 significantly enhances the energy efficiency, minimizes the operational time, and is advantageous for large-scale production. Catalyst efficiency: Method 7 employs the Sn(Oct)_2_ catalyst, which was demonstrated to be effective in Methods 3 and 4. The catalyst was also more efficient and stable than the zinc acetate dihydrate catalyst used in Method 5 and the methanesulfonic acid (MSA) employed in Method 6. The Sn(Oct)_2_ catalyst is compatible with, and soluble in, molten glycolide monomers within a temperature range of 125–135 °C. Following the mixing stage, the oxygen atoms in the glycolide monomers form coordination bonds with the tin atoms in Sn(Oct)_2_. At this juncture, compounds containing active hydrogen initiate the ROP through nucleophilic attack, leading to an electronic rearrangement of the monomers and their subsequent insertion into the Sn–O bond, thus completing the polymerization process [23]. Notably, Sn(Oct)_2_ has been approved by the European Union as a food additive, which provides a further rationale for the adoption of this catalyst in this synthesis study [48,49]. Product quality and application prospects: The PGA synthesized via Method 7 exhibits superior crystallinity and thermal properties than PGA prepared using other methods, indicating that its degradation rate and mechanical strength are easier to control. Furthermore, the mild reaction conditions are conducive to the synthesis of high-purity, consistent products through fine chemical processes. In conclusion, Method 7 performs the best in terms of thermal properties, reaction conditions, catalyst efficiency, and product quality, establishing it as the most suitable approach for PGA synthesis. Method 8 was developed by incorporating GO into the PGA matrix following the ROP process of Method 7. The presence of GO further increased the melting temperature and crystallinity, with the highest crystallinity of 79% observed at 2 wt% GO. This enhancement can be attributed to the heterogeneous nucleation effect of GO, which provides abundant surface functional groups for hydrogen-bonding interactions with PGA chains, thereby promoting molecular chain alignment and facilitating the formation of highly ordered crystalline domains. These results indicate that Method 8 not only retains the advantages of Method 7 but also produces PGA with superior thermal and structural properties, demonstrating its potential for advanced biomedical and high-performance engineering applications.

## 4. Conclusions

This study systematically investigated the isothermal crystallization behavior of high-molecular-weight PGA synthesized via ring-opening polymerization. The DSC and kinetic analyses showed that crystallization proceeds faster at higher temperatures but results in lower final crystallinity, whereas lower temperatures lead to slower crystallization with higher crystallinity. Avrami exponents in the range of 1.47–1.51 confirmed that heterogeneous nucleation and lamellar crystal growth dominate the crystallization process. The PGA exhibited its highest crystallinity within the narrow crystallization window of 176–177 °C, thereby highlighting the importance of precise temperature control. Overall, these findings clarify the relationship between the temperature, crystallization kinetics, and crystalline structure of PGA, and demonstrate that temperature optimization combined with the addition of GO is an effective strategy for tailoring its properties for biomedical and engineering applications. The incorporation of GO further enhanced the crystallization performance of PGA. Acting as an efficient heterogeneous nucleating agent, GO promoted chain alignment and increased both the melting temperature and crystallinity, with 2 wt% GO identified as the optimal loading for achieving the highest crystallinity.

## Figures and Tables

**Table 1:** Summary of final crystallinity: Avrami index (*n*), crystallization rate constant (*K_c_*), and half crystallization time (*t*_1*/*2_) of PGA sample at varying crystallization temperatures (*T_c_*).

Number	T_c_ (°C)	Final Crystallinity ^a^ (%)	*n * ^b^	*K*_c_ ^c^ (min^−n^)	*t*_1/2_ ^d^ (s)
A	175	68	1.47	6.3 × 10^−4^	111
B	176	70	1.50	5.6 × 10^−4^	115
C	176.5	74	1.51	5.6 × 10^−4^	112
D	177	77	1.48	6.1 × 10^−4^	116
E	177.5	68	1.41	9.0 × 10^−4^	111

^a^ Final Crystallinity (%): This parameter represents the percentage of crystallinity achieved by a sample at a specific crystallization temperature. It can be determined using differential scanning calorimetry (DSC). ^b^ Calculated Using the Avrami Equation: The crystallinity fraction *X*(*t*) at time *t* is calculated using the Avrami equation, *X*(*t*) = 1 − *exp*(*−Kt^n^*), where K is the crystallization rate constant, and *n* is the Avrami exponent. The Avrami fittings exhibited high statistical confidence, with regression coefficients (R^2^) above 0.99 across all temperatures. ^c^ Crystallization Rate Constant (*K_c_*): This constant is typically derived by fitting experimental data to the Avrami equation. It reflects the speed of the crystallization process. ^d^ Half-Crystallization Time: This is the time required for a sample to reach 50% of its final crystallinity. For certain crystallization models, it can be related to *K* and *n* and is calculated using the formula, t_1/2_ = [(ln2)/K]^1/n^.

**Table 2:** Enthalpies of fusion and endothermic peaks of test samples prepared at varying isothermal crystallization temperatures.

Number	*T_c_* (°C)	GO (wt%)	*T_m_* (°C)	ΔH*_f_* (J g^−1^)
A	175	0	*T_m_*_1_ = 206.2 ± 2.8	32.00 ± 4.2
			*T_m_*_2_ = 218.6 ± 2.4	44.94 ± 3.8
B	176	0	*T_m_*_1_ = 208.8 ± 3.2	35.08 ± 2.3
			*T_m_*_2_= 219.2 ± 3.0	45.16 ± 2.4
C	176.5	0	*T_m_*_1_ = 207.1 ± 1.2	37.25 ± 5.6
			*T_m_*_2_ = 219.0 ± 1.6	49.26 ± 4.9
D	177	0	*T_m_*_1_ = 206.3 ± 2.1	38.18 ± 3.2
			*T_m_*_2_ = 218.1 ± 2.3	49.78 ± 3.8
E	177.5	0	*T_m_*_1_ = 205.3 ± 1.5	36.31 ± 4.1
			*T_m_*_2_ = 217.3 ± 1.6	51.75 ± 3.5
F	177	1	*T_m_*_1_ = 207.2 ± 1.2	37.23 ± 2.3
			*T_m_*_2_ = 219.1 ± 1.4	51.36 ± 2.8
G	177	2	*T_m_*_1_ = 209.4 ± 1.3	38.46 ± 1.5
			*T_m_*_2_ = 220.0 ± 1.6	52.18 ± 2.6
H	177	3	*T_m_*_1_ = 208.1 ± 2.1	36.12 ± 3.2
			*T_m_*_2_ = 218.7 ± 2.3	51.35 ± 2.3

**Table 3:** Summary of PGA synthetic methodologies and corresponding thermal properties.

Number	Synthesis Methodology	Reaction Conditions	Reaction Time	Catalyst	T_m_ (°C)	Crystallinity (%)	Ref.
1	Melt/solid polycondensation	190 °C, 150 ⟶ 30 (mmHg) 190 °C (N_2_ system)	5 h (liq. Oligomer) 20 h (powder)	SnCl_2_ ^d^	-	40	[50]
2	Melt/solid polycondensation	190 °C, 150 ⟶ 30 (mmHg) Melting at 230 °C, cooling to 190 °C	5 h (liq. Oligomer) 20 h	SnCl_2_ ^d^	-	39	[50]
3	Melt ROP ^b^ (1-dodecanol initiator)	Melting monomers at 95 °C 210/235 °C	-	Sn(Oct)_2_	222	-	[48]
4	Melt ROP ^b^ (1,4-butandiol initiator)	Melting monomers at 95 °C 210/235 °C	-	Sn(Oct)_2_	222	-	[48]
5	Melt/solid polycondensation	220 °C, 150 ⟶ 100 ⟶ 30 (Torr)	7 h	Zinc acetate dihydrate	229	51	[51]
6	Solution polymerization (diphenylsulfone solvent)	170–200 °C, 100 (Torr)	20 h	MSA ^a^	229	-	[23]
7	ROP ^b^	130–140 °C, 100 (Torr)	12 h	Sn(Oct)_2_ ^e^	218	68–77	This study
8	ROP with GO (2 wt%) ^b,c^	130–140 °C, 100 torr, GO dispersed in HFIP and added to PGA prior to drying ^c^	12 h	Sn(Oct)_2_ ^e^	220	79	This study

^a^ MSA: methanesulfonic acid. ^b^ ROP: ring-opening polymerization. ^c^ GO: graphene oxide. ^d^ SnCl_2_: stannous chloride. ^e^ Sn(Oct)_2_: Tin(II) 2-ethylhexanoate.

## Data Availability

The original contributions presented in this study are included in the article. Further inquiries can be directed to the corresponding authors.
